# Ethosomes of Phenylethyl Resorcinol as Vesicular Delivery System for Skin Lightening Applications

**DOI:** 10.1155/2017/8310979

**Published:** 2017-07-19

**Authors:** Tunyaluk Limsuwan, Prapaporn Boonme, Pasarat Khongkow, Thanaporn Amnuaikit

**Affiliations:** ^1^Department of Pharmaceutical Technology, Faculty of Pharmaceutical Sciences, Prince of Songkla University, Songkhla 90112, Thailand; ^2^Nanotec-PSU Center of Excellence on Drug Delivery System, Songkhla, Thailand; ^3^Institute of Biomedical Engineering, Faculty of Medicine, Prince of Songkla University, Songkhla 90112, Thailand

## Abstract

Ethosome formulations containing phenylethyl resorcinol (PR) were developed. The formulation was produced from 0.5% w/v PR, 0.5% w/v cholesterol from lanolin, 3% w/v L-*α*-phosphatidylcholine from soybean, 30% v/v absolute ethanol, and water up to 100% v/v. It was characterized by a vesicular size of 389 nm, low polydispersity index of 0.266, zeta potential of −34.19 ± 0.44 mV, high PR entrapment efficiency of 71%, and good stability on storage at 4 and 30°C at 75% RH for 4 months. In vitro studies using pig skin revealed that permeation coefficient of PR from ethosomes was significantly higher than that from liposomes. In vitro retention profiles showed that PR accumulation in pig skin following application of ethosome formulations was 7.4-, 3.3-, and 1.8-fold higher than that achieved using liposomes, 20% propylene glycol solution, and 30% hydroethanolic solution, respectively. An inhibition value of around 80% was measured for antityrosinase activity of PR in pig skin. Consistently, ethosomes exhibited higher tyrosinase inhibition activity and melanin content reduction when compared to other formulations in B16 melanoma cells. Ethosomes did not cause acute dermal irritation in albino rabbits. These findings demonstrate that ethosomes are capable of delivering PR into the skin efficiently and hold promise for topical application of skin lightening products.

## 1. Introduction

Phenylethyl resorcinol (PR) is a phenolic compound produced from resorcinol. PR has attracted much attention as a lightening agent in skin care products [1] since it inhibits tyrosinase activity and thus the conversion of tyrosine to L-dopa. Tyrosine is the substrate of tyrosinase and oxidized to L-dopa in melanin synthesis; therefore the substance that can inhibit tyrosinase leads to skin whitening. PR has been reported to be approximately 22 and 10 times more effective than kojic acid when assessed using the in vitro mushroom tyrosinase and in vitro epidermal model (MelanoDerma™), respectively [2]. PR has been shown to lighten Asian human skin in vivo when applied topically with formulation at concentration of 0.5% [3]. However, the formulation of PR for topical application is difficult because of the poor aqueous solubility of the compound and its low stability when exposed to light [4], which results in a color change from white to pink tones. In addition, concentrations of PR in the formulation in excess of 1% can cause skin irritation which may lead to consumer rejection of the product [5].

Ethosomes are vesicular systems produced by combinations of phospholipid, ethanol, and water. The high ethanol concentration results in a highly elastic vesicle membrane which is advantageous for enhanced skin delivery [6]. In addition, the presence of ethanol in the formulation encourages the formation of lamellar-shaped vesicles which have been reported to improve the solubility and entrapment of many drugs including minoxidil and testosterone [6, 7]. Ethosomes have been shown to improve the skin permeability of many drugs due to interactions between the high ethanol content of the formulation, the phospholipid presence in the vesicles membrane, and skin lipid molecules. Ethanol interacts with the polar head group of skin lipids resulting in reduced rigidity of lipid domains in the stratum corneum. The increased fluidity of the liquid crystalline state may contribute to increased skin permeability. Ethanol is also considered to increase the elasticity of ethosome vesicles such that passage through the lipid pores and channels in the stratum corneum is facilitated [8, 9]. Drug release is expected to be influenced by fusion of vesicle membranes with skin lipids at various points along the penetration pathway [6–9]. Ethosome formulations therefore offer a promising strategy for overcoming the problems of topical delivery of PR potential by increasing the solubility and light stability of the phenolic compound and decreasing skin irritation. Thus PR was encapsulated in ethosomes and the stability, in vitro skin permeation, and skin irritation were assessed to evaluate the suitability of the formulations for skin whitening applications.

## 2. Materials and Methods

### 2.1. Materials

PR was purchased from Starchem Enterprises Limited (Nanjing, China). SPC and CHOL were purchased from Sigma-Aldrich (Missouri, USA). Absolute ethanol was bought from RCI Labscan Limited (Bangkok, Thailand). Kojic acid, tyrosinase enzyme, and dimethyl sulfoxide (DMSO) were purchased from Sigma-Aldrich (WGK, Germany). Milli-Q water was used throughout the experiment. All chemicals were used as received without any modification.

### 2.2. Preparation of Ethosome Containing PR

The ethosome formulations were prepared using a thin-film hydration method. Formulations were composed of 0.5% w/v PR, 0.5% w/v CHOL, 3 and 6% w/v SPC, 10–60% v/v absolute ethanol, and water up to 100% v/v. The oil phase consisted of SPC, CHOL, and PR dissolved in absolute ethanol. The aqueous phase was a hydroethanolic solution which consisted of various ratios of ethanol and water. Both oil phase and aqueous phase were separately sonicated at 60°C for 30 minutes until being homogenous. The oil phase was added in a 500-ml round-bottomed flask and a thin lipid film was formed by removal of ethanol using a rotary evaporator (Model Eyela N-1000 series, Tokyo Rikakikai Co., Ltd., Japan). The lipid film was hydrated with 10 ml of aqueous phase, followed by shaking for 5 minutes. Each flask individual was closed tightly with Parafilm to avoid water evaporation and sonicated at 60°C for 30 minutes to obtain the final ethosome formulation. PR-ethosome colloidal liquid system of formulations with concentration 0.5 mg/ml was used for characterization and efficacy studies. Each formulation was visually inspected for homogeneous appearance without phase separation or precipitation [10] and the most suitable formulation was subjected to more detailed analysis.

### 2.3. Characterization of Ethosome Formulations Containing PR

#### 2.3.1. pH and Viscosity Evaluation

The pH of the optimized formulation was measured by using a pH meter (Mettler-Toledo, Switzerland). Viscosity was evaluated using a Brookfield DV-III Ultra Rheometer (Brookfield Engineering Laboratories Inc., USA) with a SC4-31 spindle at rotation speed of 250 rpm.

#### 2.3.2. Vesicle Size, Size Distribution, and Zeta Potential

The vesicle size, size distribution, and zeta potential of PR-containing ethosomes were determined using a Zeta Potential Analyzer (Model ZetaPALS, New York, USA) at 25°C with a scattering angle (*θ*) of 90 degrees after diluting 10 *μ*l of the vesicular formulation with 4 ml of Milli-Q water. All determinations were performed in triplicate.

#### 2.3.3. Determination of PR Entrapment Efficiency

The percent entrapment efficiency (% EE) of PR in each ethosome formulation was determined using the ultracentrifugation method. The formulation (5 g) was centrifuged in an Ultracentrifuge (Optima™L-100XP, Beckman, USA) equipped with SW60 Ti Rotor at 60,000 rpm, 4°C for 1.5 h. The supernatant was collected and diluted with Milli-Q water for determination of the amount of PR not entrapped in the lipid vesicles. For determination of the total PR amount in the formulation (oil phase and aqueous phase), the vesicles were broken down by being diluted with methanol at the sample and solvent ratio of 1 : 1 and filtered through a 0.45 *μ*m syringe filter membrane. Samples were analyzed for PR content using HPLC. All samples were analyzed in triplicate and the % EE was calculated according to the equation: (*T* − *F*)/*T* × 100%, where *T* is the total amount of PR in the formulation and *F* is the nonentrapped PR amount in the aqueous phase. The optimized ethosome composition was selected on the basis of a vesicle size less than 500 nm, narrow size distribution (pdI < 0.3), high absolute value of zeta potential, and % EE > 50% [11].

#### 2.3.4. Determination of PR Using High-Performance Liquid Chromatography (HPLC)

Quantitative determination of PR was performed using the Agilent 1100 series HPLC (Agilent Technologies, Morges, Switzerland) equipped with photodiode-array detector (PDA). The reverse-phase chromatography was carried out with a BDS HYPERSIL C_18_ column (150 × 4.6 mm, 5 *μ*m) at room temperature (25°C). The mobile phase was a mixture of acetonitrile/methanol/milli-Q water (40 : 20 : 40 v/v) and flow rate was maintained at 0.8 ml/min. The 20 *μ*l of the sample was injected and detected at 254 nm.

#### 2.3.5. Visualization of Ethosome Formulations Using Scanning Electron Microscopy

The morphology of dried optimized ethosome formulations was examined using scanning electron microscope (Quanta 400, FEI, Czech Republic) at an accelerating voltage of 20 kV. Samples were prepared according to Gram's Method, wherein 100 *μ*l of PR-ethosome formulation was diluted with 3 ml Milli-Q water, dropped onto a prior cover slip, and dried. Crystal violet solution (2-3 drops) was applied to the dry sample for about 1 minute, followed by a drop of Gram's iodine solution. The positive charge of the crystal violet stain interacted with the negative charge of the ethosome lipid vesicles and subsequently formed a complex with I^−^ and I_3_^−^ [11]. The sample was dried and coated with gold in a sputter coater (Leica EMD005, Czech Republic) under an argon atmosphere (50 Pa) at 50 mA for 70 seconds. Samples were observed at 80,000x magnification.

#### 2.3.6. Degree of Deformability

The deformability of the optimized ethosome was studied using method which was modified from Jain et al. [12]. In this study, among the ethosome vesicles through a large number of pores of known size, 5 ml of the optimized ethosome was filtered through a 200 nm polycarbonate filter with an external pressure of 8.5 × 10^−4^ bar for 5 minutes. The vesicle size and size distribution before and after filtration were measured by Zeta Potential Analyzer (Model ZetaPALS, New York, USA) at 25°C with a scattering angle (*θ*) of 90 degrees. All determinations were performed in triplicate. The degree of deformability of the optimized ethosome was calculated using equation: *D* = *J∗*(*r*_*v*_/*r*_*p*_)^2^,  where *D* is deformability of vesicle membrane, *J* is amount of ethosome, which was extruded during 5 minutes, *r*_*v*_ is size of vesicles (after passes), and *r*_*p*_ is pore size of the filters.

### 2.4. Storage Stability of PR-Ethosome Formulations

Samples of the optimized PR-ethosome formulation were sealed in glass vials, tightly capped with Parafilm to avoid evaporation, and stored at 4 ± 1°C, 30 ± 1°C, and 45 ± 1°C under control humidity of 75% RH in a Constant Climate Chamber Model HPP260 (Memmert, Schwabach, Germany) for 0, 1, 2, and 4 months. The stability of the formulations was assessed periodically at intervals of 0, 1, 2, and 4 months in terms of the physical appearance, vesicle size, size distribution, zeta potential, total PR content, and entrapment efficiency. Testing was carried out using triplicate samples at each storage condition.

### 2.5. In Vitro Skin Permeation of PR-Ethosome Formulations

The efficiency of PR delivery into skin by topical application of ethosome formulations was evaluated in vitro using a modified version of the method of Limsuwan et al. [13]. Newborn pig skin was used as the diffusion membrane in a modified Franz-diffusion cell (Model Hanson 57-6M, California, United States) with an effective diffusion area of 1.77 cm^2^. The receptor medium was a mixture of 0.44% w/v sodium chloride (NaCl) in phosphate buffer pH 7.4 : propylene glycol (80 : 20% v/v). The receptor compartment was filled with 11 ml of receptor medium, maintained at 37 ± 1°C, to ensure a skin surface temperature of 32 ± 2°C. The receptor medium was stirred at 500 rpm throughout the experiment using a magnetic stirrer. The pig skin diffusion membrane was placed between the donor and receptor compartments with the stratum corneum toward the donor compartment. A sample of PR-ethosome formulation (1 ml) was applied to the skin surface in the donor compartment, which was then capped tightly with Parafilm. A PR-liposome, formulation which had the same composition as ethosome but without ethanol, was used as a control. A sample of receiver medium (1 ml) was withdrawn through the sampling port of the diffusion cell at 0.5, 1, 2, 4, 6, 8, 12, and 24 h time intervals. The receptor medium was immediately replenished with equal volume of fresh medium at 37 ± 1°C to maintain sink conditions. The experiments were repeated five times and samples of receiver medium were analyzed in triplicate for PR content by HPLC.

The cumulative amount (*Q*_*t*_, *μ*g/cm^2^) of PR that permeated through the skin membrane was plotted as a function of time. The steady state flux (*J*_ss_) was calculated from the slope of the linear portion of the plot. The lag time (*T*_lag_) of the PR to permeate through the pig skin before reaching the receptor fluid was calculated from the X-intercept of the plot. The value of permeation coefficient (*K*_*p*_) for PR was calculated using the equation: *K*_*p*_ = *J*_ss_/*C*_0_, where *C*_0_ is the initial concentration of PR in the donor compartment.

On termination of the experiment at 24 h, the excess PR-ethosome formulation was collected from the skin surface and the membrane was cleaned by wiping with a cotton ball soaked in 10 ml of methanol. The sample was sonicated at room temperature (32 ± 2°C) for 30 minutes and centrifuged using a Hermle Z323K Centrifuge (Wehingen, Germany) at 6,000 rpm, 4°C for 30 minutes. The clear supernatant was collected and lysed with methanol in the ratio of 1 : 1 (v/v). The sample was filtered through 0.45 *μ*m nylon membrane and analyzed using HPLC to determine the amount of residual PR in the donor compartment. All samples were analyzed in triplicate.

### 2.6. Retention of PR in Skin Diffusion Membrane

Following removal of the ethosome formulation from the donor compartment, the surface of the skin diffusion membrane was retrieved, cleaned with methanol, and cut into small pieces with a pair of surgical scissors. The skin fragments were homogenized (Model PT 1200 E, Polytron, Switzerland) at 24,000 rpm in 5 ml methanol at room temperature (32 ± 2°C) for 2 minutes to extract the active. Subsequently, the sample was sonicated at room temperature for 30 minutes and centrifuged using a Hermle Z323K Centrifuge (Wehingen, Germany) at 6,000 rpm at 4°C for 30 minutes to separate the skin lipid. The clear supernatant was collected, filtered through 0.45 *μ*m nylon membrane, and analyzed using HPLC to determine the amount of PR retained in the skin and all samples were analyzed in triplicate.

### 2.7. Tyrosinase Inhibition Assay

The PR which had accumulated in the skin diffusion membrane after permeation testing of PG solution, liposome, and ethosome formulations for 24 h was assayed for antityrosinase activity. These samples were extracted from pig skin fragments as described above (Section 2.6). Antityrosinase activity was measured using the dopachrome method. Dopachrome is one intermediate substrate in melanogenesis; this method used the L-dopa as a substrate. The oxidation of L-dopa can be converted to dopachrome, which showed red color and can be detected by visible light at 492 nm [14]. Kojic acid and* Artocarpus lakoocha* water extract were used as positive controls [14]. In brief, 20 *μ*l of test samples or positive controls in DMSO were mixed with 140 *μ*l phosphate buffer pH 6.8 (PBS) and 20 *μ*l tyrosinase solution (203.3 unit/ml) in 96-well microplates. After 10 minutes, 20 *μ*l of 0.85 mM L-dopa was added to all wells and the optical density (OD) of the reaction at 492 nm was measured as the OD before incubation. The microplate was subsequently incubated at 25°C for 20 minutes and the OD of the reaction was recorded as after incubation. Following incubation, the amount of dopachrome was produced in the reaction mixture.

The antityrosinase activity was expressed in terms of % tyrosinase inhibition, calculated according to the equation: [Activity = (*A* − *B*)−(*C* − *D*)/(*A* − *B*)] × 100%, where *A* is the difference of OD before and after incubation at 492 nm (containing all reagents and enzyme except the test sample), *B* is the difference of OD before and after incubation at 492 nm (containing all reagents except the test sample and enzyme), *C* is the difference of OD before and after incubation at 492 nm (containing all reagents, test sample, and enzyme), and *D* is the difference of OD before and after incubation at 492 nm (containing all reagents and the test sample except enzyme).

### 2.8. Cell Culture

B16 melanoma cell line was a gift kindly provided by Assist Professor Dr. Sukanya Dej-adisai, in the Faculty of Pharmaceutical Sciences, Prince of Songkla University (Thailand). Cells were cultured in Dulbecco's modified Eagle's medium (DMEM), supplemented with 10% fetal bovine serum (Gibco, USA), 2 mM glutamine, and 100 units/ml penicillin/streptomycin, and maintained at 37°C in a humidified incubator with 10% CO_2_.

### 2.9. Quantitative Assay of Tyrosinase Activity in B16 Melanoma Cells

Tyrosinase activity was performed by adapting the method described by Tomita et al. [15] with slight modification. Briefly, cells (5 × 10^4^ cells/well) were incubated in 24-well plates with different formulations containing 5 *μ*M of PR. After treatment, the cells were washed twice with phosphate buffered saline (PBS) and freeze-thaw lysed in 200 *μ*L of 2% Triton X-100 in 0.1 M phosphate buffered saline (pH 7.2). After protein quantification by BCA method, 100 *μ*L of cell lytic solution (100 *μ*g/mL) was mixed with 100 *μ*L of 0.2% L-dopa in phosphate buffer solution (pH 7.2) and incubated for 1 hour at 37°C. The absorbance was measured at 475 nm, using control cells as 100%, and the dopachrome formations of each sample were compared.

### 2.10. Quantitative Assay of Melanin Content in B16 Melanoma Cells

Cells (5 × 10^5^ cells/dish) were incubated in 60 mm dishes with different formulations containing 5 *μ*M of PR. After treatment, the cells were washed twice with PBS and lysed in 200 *μ*L of 1 N NaOH for 1 h at 95°C to solubilize the melanin. Lytic solution (100 *μ*L) was added in a 96-well plate. The total amount of melanin was measured at 405 nm. The melanin content was calculated and adjusted with the concentrations of proteins, using control cells as 100%.

### 2.11. Acute Dermal Irritation Test of PR-Ethosome Formulations

The acute irritation/corrosion properties of PR-ethosome formulations were assessed in rabbits by the Thailand Institute of Scientific and Technology Research (TISTR) according to the Test Guideline (TG) number 404 of the OECD Guidelines for Testing of Chemicals (2002). Briefly, three healthy adult albino rabbits (New Zealand white hybrid strain, body weight range 2-3 kg) were purchased from the Department of Animal Science, Faculty of Agriculture, Kasetsart University, Thailand. One day before experimentation, the epidermal hair of the dorsolumbar region (approximately 10 × 10 cm^2^) was removed and two areas of the shaven skin (approximately 2.5 × 2.5 cm^2^) were selected. PR-ethosome formulation (0.5 ml) was applied to a gauze patch size while 0.5 ml of distilled water was applied to a second patch as a negative control. Both gauze samples were applied to the selected skin sites of each rabbit and secured with adhesive tape to avoid movement. After 4 h, both samples were removed and the treated skin was wiped gently with moistened cotton wool to remove any residual test material. An irritation of skin was observed by the level of erythema and oedema at time 1, 24, 48, and 72 h or more than these time intervals when the irritation still occurred, but not over 14 days of observation periods. The PR hydroethanolic solution was used as a positive control. The skin reactions were scored accounting for the numerical scoring system from 0 to 4, relating to none to severe signs by two professional inspectors.

### 2.12. Statistical Analysis

Results are presented as mean ± standard deviation (SD) or mean ± standard error of mean (SEM). One-way analysis of variance (ANOVA) followed by post hoc analysis was used to test the statistical significant of differences among groups. *P* < 0.05 was considered statistically significant.

## 3. Results and Discussion

### 3.1. Formulation and Characterization of PR-Ethosome Formulations

Ethosome formulations containing 3 and 6% w/v SPC in combination with 20–40% v/v ethanol exhibited good physical appearance due to the enhanced PR solubility in the ethanol : water as cosolvent system and lipid vesicles (4.83 mg/ml in 10% ethanol and 0.26 mg/ml in water of PR solubility, Limsuwan et al. [10]). The concentration of ethanol in the formulation was found to be a major factor in colloid formation. The inclusion of 10% v/v ethanol was insufficient to solubilize the active whereas formulations containing in excess of 50% v/v ethanol resulted in precipitation due to disruption of the lipid vesicles [10].

#### 3.1.1. pH and Viscosity of PR-Ethosome Formulations

The viscosity of all ethosome was found in the range of 25.43 ± 0.25 and 88.67 ± 0.47 centipoise (cps). The viscosity of the optimized ethosome was found to be 31.73 ± 0.21 cps. The pH of the optimized formulation was 5.05 ± 0.01 which is close to pH of the normal skin (pH 4.5–5.5).

#### 3.1.2. Vesicle Size and Size Distribution

The size of ethosomes was found in the range of 214 to 890 nm. The smallest vesicles (214 and 353 nm) were produced in formulations containing 3% w/v SPC in combination with 40 and 50% v/v ethanol, respectively. All other formulations may be considered to be outside the nanosize range. Increasing the phospholipid concentration from 3–6% SPC resulted in a significant increase in vesicle size range from 214–395 nm to 338–893 nm due to the increase of phospholipid molecules in the vesicle bilayers where PR resided. In contrast, increasing ethanol concentration in the formulation resulted in a decrease in vesicle size. For example, ethosome formulations containing 40% v/v ethanol (338.1 ± 4.1 nm) exhibited a vesicle size of 338 nm compared with 893 nm in formulations containing 10% v/v ethanol and an equivalent phospholipid concentration (6% w/v SPC). The smallest vesicles were observed in formulations containing 40% v/v ethanol in both the 3% w/v SPC (213.7 nm) and 6% w/v SPC systems (338.1 nm). The decrease in ethosome size with increasing ethanol concentration is considered to result from an interpenetration of a hydrocarbon chain (ethanol) in the vesicle lipid bilayer, leading to a decrease in vesicle size [16–19]. In addition, Rakesh and Anoop [20] explained that the high ethanol concentration may provide a net negative charge to the ethosome system and confers it some degree of steric stabilization that may finally lead to decrease in ethosome size. The pdI of all ethosome formulations was less than 0.3, indicating a narrow vesicle size distribution and high degree of homogeneity [18].

#### 3.1.3. Zeta Potential

The zeta potential values of all ethosome formulations were highly negative and generally above −20 mV due to the presence of ethanol in the system which may provide the negative charge on vesicles surface in the range −20.9 to −35.6 mV. Using ethanol in the range of 20–40% v/v gave mostly higher zeta potential value than that in the low (10% v/v) and high (50 and 60% v/v) concentrations with 3% w/v SPC producing zeta potential values around −22 mV and correlating with poor formulation stability (Table 1). Precipitation occurred soon after preparation for formulations containing 10 and 60% ethanol and after 1 week in the case of formulations prepared using 50% v/v ethanol. Higher zeta potentials of −33.1 to −35.6 mV were measured for ethosome formulations containing 20–40% v/v ethanol and 3% SPC, indicating higher stability [21, 22]. The higher stability of these formulations is attributed to modification of the net negative surface charge in the presence of ethanol thereby enhancing charge stabilization of the vesicles due to electrostatic repulsion and delaying the formation of aggregates [17].

#### 3.1.4. PR Entrapment Efficiency of Ethosome Formulations

**Figure 1:**
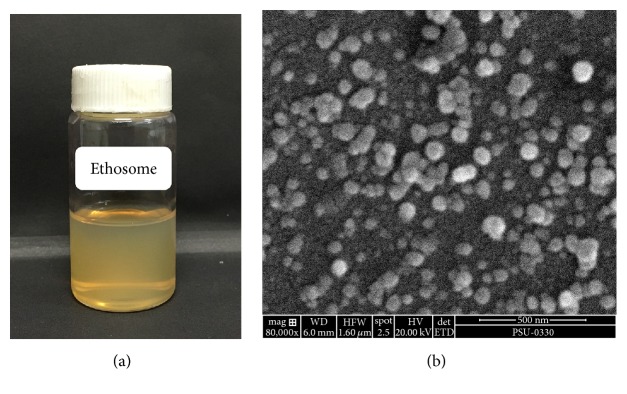
The physical appearance (a) and scanning electron micrograph (b) of the optimized ethosome formulation containing 0.5% w/v PR, 0.5% w/v CHOL, 3% w/v SPC, 30% v/v absolute ethanol, and water up to 100% v/v.

The amount of drug incorporated in the ethosome formulation influences the kinetics of delivery of the active through or into the skin, the stability of the entrapped active in the formulation, and skin irritation. The effects of ethanol and phospholipids concentrations on PR entrapment efficiency in the ethosome formulations are shown in Table 1. The percent entrapment efficiency (% EE) of PR in ethosome formulations ranged from 4.6 to 71%. EE was found to increase with increasing ethanol concentration from 10 to 30% v/v but decreased dramatically at higher ethanol concentrations similar to the findings of Maurya [18]. The formulation prepared from 3% w/v SPC with 30% ethanol showed the highest EE (71%). This behavior may be explained by the presence of ethanol in the vesicles which increases PR solubility in both the core and membrane. However, increasing ethanol concentrations above 40% v/v led to a marked reduction in PR entrapment which may be due to membrane leakage effects due to the presence of ethanol [6]. Bhalaria et al. [23] reported that the EE of fluconazole increased with an increase in concentration of ethanol in ethosome formulations from 10 to 30% v/v. In our study, it was similarly found that the EE increased significantly with an increase in concentration of ethanol in the ethosome formulation and with the further increasing ethanol concentration more than 30% v/v led to decrease of EE (Table 1), which may be explained by the vesicle membrane which becomes more permeable and leakage. The optimized ethosome composition in this study was based on a vesicle size < 500 nm, pdI < 0.3, high zeta potential, and EE > 50%. The application of PR as skin product is limited from the poor water solubility, instability in light, and skin irritation. The optimized ethosome formulation focused on high entrapment efficiency since this would decrease skin irritation by free PR, reduce the chance of photo-degradation of the active, and improve penetration and permeation of PR through the skin. Therefore, the ethosome formulation containing 0.5% w/v PR, 0.5% w/v CHOL, 3% w/v SPC, 30% v/v absolute ethanol, and water up to 100% v/v was selected as the optimum system. This formulation was characterized by a vesicle size of 389 nm, a low pdI of 0.266, zeta potential of −34.2 mV, and EE of 71%. The physical appearance was yellowish colloidal (Figure 1(a)) and scanning electron micrographs revealed spherical vesicles as shown in Figure 1(b).

#### 3.1.5. Degree of Deformity of PR-Ethosome Formulations

The deformability test of the optimized ethosome and the size of the vesicles before filter through a 200 nm polycarbonate filters was 363.67 ± 22.38 nm with pdI being 0.22 ± 0.05. After filtration, the size of the ethosome vesicles was 202.63 ± 2.26 nm. The pdI was 0.20 ± 0.08. The amount of ethosome which was extruded during 5 minutes was 3.23 ± 0.25 ml. The degree of deformability of ethosome was 3.33 ± 0.19. However, the liposome with the vesicle size 600.23 ± 11.92 nm cannot pass 200 nm pore size. This characteristic could be explained by high ethanol concentration present in ethosome formulation; it reduced the interfacial tension of the vesicle membrane leading to providing elasticity of the vesicle membrane. In contrast, the liposome formulation is rigid vesicle [9]. Therefore, these results indicate that ethosome possesses a flexible membrane and can penetrate pores much smaller than their diameter.

### 3.2. Storage Stability of PR-Ethosome Formulations

**Figure 2:**
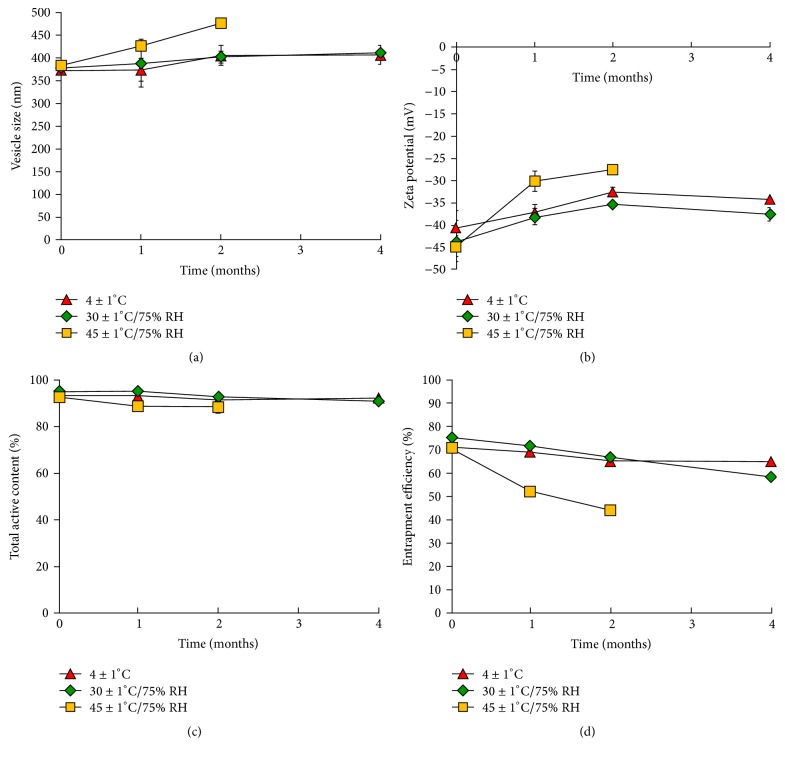
The effect of storage conditions on vesicle size (a), zeta potential (b), total active content (c), and entrapment efficiency (d) of the optimized ethosome formulation.

The vesicle size, zeta potential, change in total active content, and EE of the optimized ethosome formulation following storage at 4 ± 1°C, 30 ± 1°C, and 45 ± 1°C under controlled humidity of 75% RH for 0, 1, 2, and 4 months are shown in Figure 2. The physical appearance of the formulation (yellowish colloidal) remained unchanged of at 4°C and 30°C over 4 months. High zeta potentials above 30 mV were measured at 4°C and 30°C over 4 months demonstrating the good stability of the formulation. The vesicle size increased by around 9% but the pdIs indicated a narrow size distribution (pdI < 0.03). The increase in ethosome size might be caused by the aggregation and fusion of the ethosome itself. In addition, in the ethosome formulations, ethanol might displace to the water molecules which hydrated the phospholipid head group and served as a repulsive force (hydration force) between adjacent bilayers. When the hydration force became weak, the vesicles would initiate aggregation [24].

The reduction in active content and the EE of the formulation did not change over the 4-month storage period. A reduction in active content from 93% to 91% occurred at 4°C while the EE decreased from 71 to 65%. These stability data indicated that the optimized ethosome formulation was highly stable at 4°C and 30°C, due to the high negative net charge, leading to charge stabilization, which prevents the formation of vesicle aggregates [17]. In addition, the vesicle structure is highly efficient at storing and retaining PR molecules.

When the ethosome formulation was stored at 45°C, the EE decreased significantly from 71 to around 50% suggesting an effect from combination of the gel-to-liquid transition of the lipid components together with possible chemical degradation of the phospholipids. This causes leakage of the vesicle membrane, facilitates diffusion of PR from the vesicles, decreases drug entrapment, and finally leads to precipitation of the formulation [19, 25]. In the present study, instability of the ethosome formulation on storage at high temperature (45°C) for 4 months was indicated by precipitation of active and/or other components and a vesicle size increase of around 24% in 2 months. The zeta potential decreased to −27.5 mV at 2 months which tended to cause precipitation of the formulation. The active content and the EE of PR in the ethosome formulation decreased to 88 and 44% in 2 months, respectively. Therefore, the ethosome formulation prepared in this study needs to be stored at 4°C and 30°C.

### 3.3. In Vitro Skin Permeation and Skin Retention Studies

The amount of PR in the three compartments of the diffusion cell (donor compartment, skin diffusion membrane, and receptor compartment) following application of ethosome, liposome, PG solution, and 30% hydroethanolic solution formulations was analyzed following experimentation for 24 h. The detected PR in each part compartment was expressed as a percentage of the applied dose (0.5 mg/ml). The total PR recovery for all formulations ranged from 80 to 110% which is within acceptable limits of ICH guidelines [26] and demonstrates the performance and reliability of the method. Following application of the PR formulations to the pig skin diffusion membrane, most of the PR remained in the donor compartment. The low penetration of PR into the skin membrane is explained by the barrier properties of the outermost skin layer, that is, the stratum corneum, which consists of 10 to 25 layers of dead, horny, protein-rich corneocytes embedded in a lipid matrix low [27]. However, the lowest amount of PR in the donor compartment was found in the case of PR-ethosome formulations (*P* < 0.05) which indicates a higher efficiency of delivery compared with liposomes, PG solution, and 30% hydroethanolic solution.

**Figure 3:**
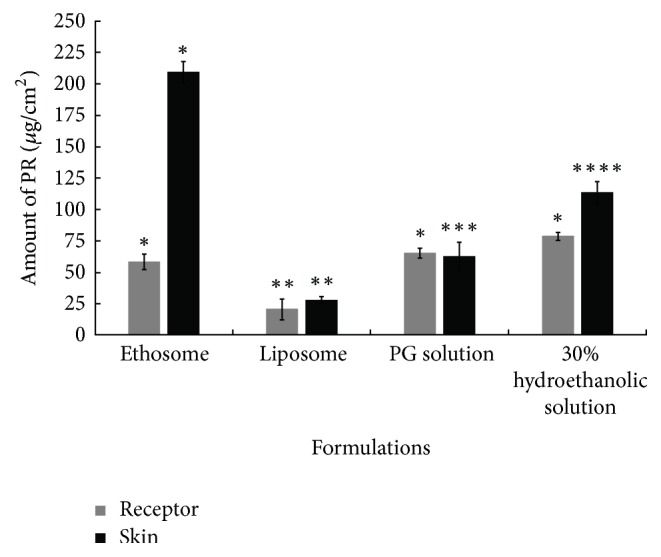
In vitro accumulation of PR in full-thickness newborn pig skin and receptor compartment after testing ethosome, liposome, 20% PG solution, and 30% hydroethanolic formulations for 24 h. Each bar represents the mean ± SEM (*n* = 5). *∗*–*∗∗∗∗*: means in the same color bar with different asterisk differ significantly (*P* < 0.05).

**Figure 4:**
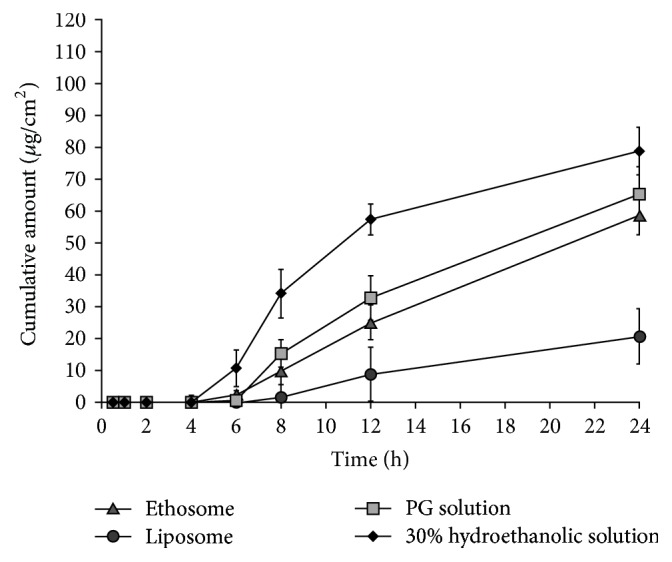
Cumulative permeation of PR across pig skin membranes following application of ethosome, liposome, 20% PG solution, and 30% hydroethanolic solution formulations. Each point represents the mean ± SEM (*n* = 5).

The accumulation and permeation of PR in pig skin diffusion membranes following application of ethosome, liposome, 20% PG, and 30% ethanolic solution formulations are shown in Figures 3 and 4 and Table 2. In case of 20% propylene glycol solution (PG solution) which represents the marketed formulation, the skin permeation profile (Figure 4) showed that PR penetrates through the skin and enters the receptor medium 5 h after application (*T*_lag_  5.0 ± 0.5 h). Moreover, the detected amount of PR in the pig skin membrane and receptor compartment was almost equivalent (Figure 3). These results demonstrate that the topical application of PR in PG solution formulations does not encourage accumulation of PR in the skin at target sites for tyrosinase inhibitor. In the case of liposomes, PR tended to remain in the donor compartment rather than the skin membrane and receptor compartment (Table 2). In addition, the skin accumulation (Figure 3) and permeation (Figure 4) of PR was lower after topical administration of liposomes compared with 20% PG solution, ethosomes, and 30% hydroethanolic solution (*P* < 0.05), demonstrating impaired skin penetration. The highest amount of PR in the receptor compartment was found following topical application of PR formulations in 30% hydroethanolic solution (Figure 3). The *Q*_24_ of PR of 30% hydroethanolic solution was 1.2-fold higher than that PG solution, 1.3-fold higher than ethosomes, and 3.8-fold higher than liposome (*P* < 0.05). In addition, the *J*_ss_ and *K*_*p*_ measured for 30% hydroethanolic solutions of PR were higher than the 20% PG solution, ethosomes, and liposomes. The high permeation of PR due to application in form of hydroethanolic solution may be due to a combination of ethanol effects involving enhanced PR solubility in the formulation, changes in the stratum corneum's barrier property, and increases in thermodynamic activity due to evaporation of ethanol [28, 29]. The effect of the phospholipid vesicles alone, ethanol, and ethanol/phospholipid combinations was investigated using liposomes, 30% hydroethanolic solution, and ethosomes (30% v/v ethanol), respectively.  Table 3 shows in vitro skin permeation parameters of PR from the ethosome, liposome, PG solution, and 30% hydroethanolic solution. Ethosomes resulted in a *K*_*p*_ value of 0.52 ± 0.05 × 10^−3^ cm/h which was significantly higher than liposomes (0.19 ± 0.08 × 10^−3^ cm/h) (*P* < 0.05), demonstrating that phospholipid vesicles alone are not sufficient to deliver PR efficiently into or through the skin. In the model developed by Touitou et al. [6], the stratum corneum lipids at physiological temperature are densely packed and highly ordered. Ethanol interacts with the polar head group region of the lipid molecules in the skin increasing fluidity and permeability. Penetration of the ethosome vesicles into the deep skin layers is facilitated through disordered stratum corneum and drug release may occur by fusion of ethosome vesicles with skin lipids along the penetration pathway [6].

The amount of PR that permeated through the skin diffusion membrane in 24 h from ethosomes and 30% hydroethanolic solution was 58.7 and 78.8 *μ*g/cm^2^, respectively (Figure 4). However, in Figure 4, it could be noted that the amount of PR that accumulated in the skin following topical application of ethosome formulations was significantly higher (209 *μ*g/cm^2^) than 30% hydroethanolic solution (113.5 *μ*g/cm^2^) (*P* < 0.05), indicating that hydroethanolic solution enhanced skin permeation of PR but not retention. Low depot formation ability of hydroethanolic solution formulations may be due to ethanol-induced skin lipid hydrolysis, leading to an increase in PR permeation into the receptor medium [30]. The *T*_lag_ of 30% hydroethanolic solution was 4.0 h which was the lowest of the delivery systems investigated.

The PR-ethosome formulations resulted in PR retention in the skin which was 7.4-, 3.3-, and 1.8-fold higher than liposomes, 20% PG solution, and 30% hydroethanolic solution, respectively (*P* < 0.05). In addition, the ratio of PR that accumulated in the skin to that in the receptor compartment at 24 h was 3.57 for ethosomes formulations, which was significantly higher than liposome (1.4), 20% PG solution (1.0), and 30% hydroethanolic solution (1.4), respectively (*P* < 0.05). Moreover, in Table 3, the *T*_lag_ of ethosomes (6.0 ± 0.6 h) was also slower than 20% PG solution (5.0 ± 0.5 h) and 30% hydroethanolic solution (4.0 ± 0.4 h). These findings indicate that ethosome formulations not only increase skin permeation but also establish PR depot formation in the skin. This behavior may arise from a combination of effects exerted by ethanol and the phospholipid component. Ethanol acts as a skin enhancer by temporarily disturbing the stratum corneum organization and increasing lipid fluidity to facilitate ethosome penetration. At the same time, the phospholipid component of the ethosome may retain the PR in the skin tissues for longer periods by vesicle fusion with skin lipids as a depot reservoir [29].

### 3.4. Antityrosinase Activity

The tyrosinase inhibitory activity of PR extracted from pig skin fragment as described in Section 2.7 is shown in Table 4. PR formulated in both 20% PG solution and ethosomes displayed high tyrosinase inhibition activity in excess of 95%, which was greater than kojic acid and equivalent to* Artocarpus lakoocha* water extract, a potent tyrosinase inhibitor [14]. The skin accumulation study (Figure 4) revealed that the ethosome formulation resulted in higher PR retention in the skin compared with the 20% PG solution formulation but tyrosinase inhibitory activity was similar. This finding suggests that the ethosomes interfere with PR activity by a shielding effect. However, formulation of PR in ethosomes, liposomes, and PG solution in pig skin showed tyrosinase inhibition activity of approximately 80% which is expected to lead to highly efficient skin lightening behavior.

### 3.5. Effects of Ethosomes of Phenylethyl Resorcinol on Melanin Production and Tyrosinase Activity

**Figure 5:**
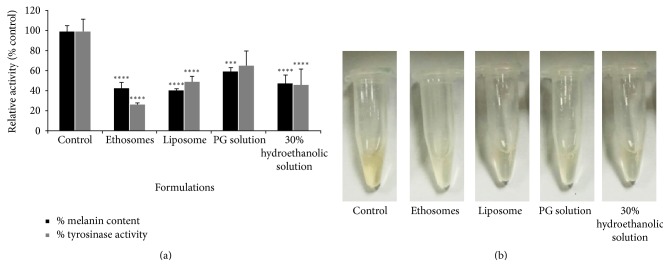
The effects of ethosomes containing PR on melanin production and tyrosinase activity in B16 melanoma cells. B16 melanoma cells were treated indicating formulations (each formulation containing 5 *μ*M PR). Relative melanin contents and tyrosinase inhibition activity were measured at 24 h after treatment. Each value represents the mean ± SD (*n* = 3). ^*∗∗∗∗*^*P* < 0.01, ^*∗∗∗*^*P* < 0.05 compared with the control (a). The visual examination of melanin content (b).

B16 melanoma cells are well-established model for melanogenic inhibitors discovery as previous studies suggested. To further evaluate impacts of ethosome containing PR on melanin production and tyrosinase activity at the cellular level, we therefore performed the experiment in B16 melanoma cells as described in Sections 2.9 and 2.10.  Figure 5 shows that PR formulated in ethosome, liposome, and 30% hydroethanolic solution significantly inhibited the tyrosinase activity as well as melanin production in melanoma cells compared with the control. Interestingly, the ethosome formulation exhibited the highest tyrosinase inhibition activity, approximately 75% inhibition, among other formulations. These findings suggest that ethosomes represent suitability for the delivery system of PR leading to the highly efficient skin lightening properties.

### 3.6. Acute Dermal Irritation Testing of PR-Ethosome Formulations

The reaction of skin to the optimized PR-ethosome formulation and PR hydroethanolic solution was evaluated using an acute irritation test in rabbits, based on erythema, eschar, and oedema formation systems. Topical application of the PR-ethosome formulation in all three test animals resulted in no erythema, eschar, and oedema formation (score = 0) in 72 h. No significant differences were recorded for animals treated with PR hydroethanolic solution (positive control). The short-term exposure suggests that PR at 0.5% was safe for application as skin products. Although ethosome provided high skin retention of PR as aforementioned, no skin irritation was found.

## 4. Conclusions

The optimized ethosome prepared by thin-film hydration method was composed of 0.5% w/v PR, 0.5% w/v CHOL, 3% w/v SPC, 30% v/v absolute ethanol, and water up to 100% v/v. This formulation had yellowish colloidal appearance with 389 nm vesicular size, low pdI of 0.266, high zeta potential of −34.19 mV, and a PR EE of 71%. It had good stability under storage stability at 4°C and 30°C (75% RH) for 4 months. In vitro skin permeation and retention profiles indicated that the ethosome formulation allowed efficient PR transport and at the same time established a depot of the active in the skin which may be advantageous for sustained antityrosinase activity. Antityrosinase activity of around 80% was measured for PR retained in pig skin at 24 h corresponding to the results in cell culture study. Ethosomes also exhibited the higher tyrosinase inhibition activity as well as the reduction of melatonin content in comparison to other formulations in B16 melanoma cells. In addition, acute dermal irritation testing in rabbits confirmed that the ethosome system did not cause skin irritation following exposure for 4 h. PR-ethosome formulations thus exhibit significant potential as skin lightening products.

## Figures and Tables

**Table 1:** The effects of ethanol and phospholipids content of ethosome formulations on physical appearance, vesicle size, pdI, zeta potential, and PR entrapment efficiency.

Composition				Physical property	Vesicle size (nm)	pdI	Zeta potential (mV)	Entrapment efficiency (%)
SPC	CHOL	Ethanol	Water					
(% w/v)		(% v/v)						
3	0.5	10	90	Crystals were observed	ND	ND	−21.22 ± 1.17	ND
		20	80	Yellow colloidal	395.1 ± 6.4	0.272 ± 0.018	−33.11 ± 1.88	64.98 ± 0.91
		*30*	*70*	*Yellow colloidal*	*388.8* ± *8.0*	*0.266* ± *0.021*	−*34.19* ± *0.44*	*71.43* ± *0.77*
		40	60	Yellow colloidal	213.7 ± 2.5	0.259 ± 0.004	−35.64 ± 1.61	41.55 ± 1.64
		50	50	Crystals were observed at 1 week	353.3 ± 2.4	0.200 ± 0.051	−22.18 ± 1.11	48.79 ± 0.19
		60	40	Yellow colloidal precipitate	ND	ND	−20.88 ± 0.97	ND

6	0.5	10	90	Yellow colloidal	893.1 ± 37.6	0.153 ± 0.064	−34.24 ± 0.75	58.26 ± 0.13
		20	80	Yellow colloidal	731.5 ± 7.2	0.251 ± 0.043	−33.47 ± 0.94	59.26 ± 0.39
		30	70	Yellow colloidal	433.9 ± 6.2	0.162 ± 0.029	−32.45 ± 1.30	66.02 ± 0.36
		40	60	Yellow colloidal	338.1 ± 4.1	0.265 ± 0.009	−34.84 ± 2.53	17.97 ± 1.08
		50	50	Yellow colloidal	755.7 ± 13.0	0.178 ± 0.055	−30.96 ± 1.02	4.56 ± 1.06
		60	40	Yellow colloidal precipitate	ND	ND	−21.38 ± 0.66	ND

Each data represents the mean ± SD (*n *= 3). ND: not detected.

**Table 2:** The percent recovery of PR from each compartment of the diffusion cell following application of ethosome, liposome, 20% PG solution, and 30% hydroethanolic solution formulations.

Formulations	Amount recovery (% of the applied dose)			
	Donor compartment	Newborn pig skin	Receptor compartment	Total
*Ethosome*	84.65 ± 3.47^a^	4.19 ± 0.17^a^	1.17 ± 0.12^a^	90.01 ± 3.59^a^
Liposome	87.51 ± 4.41^a^	0.56 ± 0.05^b^	0.41 ± 0.17^b^	88.48 ± 4.29^a^
PG solution	84.09 ± 7.46^a^	1.25 ± 0.23^c^	1.31 ± 0.17^a^	86.65 ± 7.33^a^
30% hydroethanolic solution	81.84 ± 8.86^a^	2.27 ± 0.17^d^	1.57 ± 0.15^a^	85.69 ± 8.88^a^

Data are expressed as % of total PR in the applied dose (0.5 mg/ml) (mean ± SEM, *n* = 5). a–d: means in the same column with different superscript letter differ significantly (*P* < 0.05).

**Table 3:** In vitro skin permeation parameters of PR following topical application of ethosome, liposome, 20% PG solution, and 30% hydroethanolic solution formulations.

Formulations	*J*_ss_ (*µ*g/cm^2^/h)	*K*_*p*_ (×10^−3^ cm/h)	*T*_lag_ (h)
*Ethosome*	2.60 ± 0.27^a^	0.52 ± 0.05^a^	6 ± 0.63^a^
Liposome	0.96 ± 0.42^b^	0.19 ± 0.08^b^	7 ± 1.50^a^
PG solution	2.95 ± 0.42^a,c^	0.59 ± 0.08^a,c^	5 ± 0.49^a^
30% hydroethanolic solution	3.77 ± 0.31^c^	0.75 ± 0.06^c^	4 ± 0.40^a^

Each data represents the mean ± SEM (*n *= 5). *J*_ss_, steady state flux; *K*_*p*_, permeability coefficient; *T*_lag_, lag time. a–c: means in the same column with different superscript letter differ significantly (*P* < 0.05).

**Table 4:** Tyrosinase inhibitory activity of PR in 20% PG solution, ethosome, and pig skin following topical application in 20% PG solution, ethosomes, and liposomes.

Formulations	Tyrosinase inhibition (%)
PG solution	95.54 ± 0.13
*Ethosome*	95.04 ± 0.49
PR from PG solution in pig skin	82.12 ± 0.58
PR from ethosome in pig skin	78.74 ± 0.90
PR from liposome in pig skin	79.53 ± 0.45
Kojic acid^a^	87.35 ± 0.76
*Artocarpus lakoocha* water extract^a^	96.04 ± 0.55

PR concentration in test samples: 20 *µ*g/ml. Each value represents the mean ± SD (*n* = 3). ^a^Positive control.

## References

[1] Linder J. (2014). Topical melasma treatments. *Journal of Pigmentary Disorders*.

[2] Chang T.-S. (2009). An updated review of tyrosinase inhibitors. *International Journal of Molecular Sciences*.

[3] Vielhaber G., Schmaus G., Jacobs K. (2007). 4-(1-Phenylethyl)1,3-Benzenediol: a new, highly efficient lightening agent. *International Journal of Cosmetic Science*.

[4] Fan H., Li Y., Huang Y., Liu G., Xia Q. (2014). Preparation and evaluation of phenylethyl resorcinol liposome. *Integrated Ferroelectrics*.

[5] Gohara M., Yagami A., Suzuki K. (2013). Allergic contact dermatitis caused by phenylethyl resorcinol [4-(1-phenylethyl)-1,3-benzenediol], a skin-lightening agent in cosmetics. *Contact Dermatitis*.

[6] Touitou E., Dayan N., Bergelson L., Godin B., Eliaz M. (2000). Ethosomes—novel vesicular carriers for enhanced delivery: characterization and skin penetration properties. *Journal of Controlled Release*.

[7] Fang Y., Tsai Y., Wu P., Huang Y. (2008). Comparison of 5-aminolevulinic acid-encapsulated liposome versus ethosome for skin delivery for photodynamic therapy. *International Journal of Pharmaceutics*.

[8] Verma P., Pathak K. (2010). Therapeutic and cosmeceutical potential of ethosomes: an overview. *Journal of Advanced Pharmaceutical Technology and Research*.

[9] Jain S., Tiwary A. K., Sapra B., Jain N. K. (2007). Formulation and evaluation of ethosomes for transdermal delivery of lamivudine. *AAPS Pharmaceutical Sciences and Technology*.

[10] Limsuwan T., Boonme P., Amnuaikit T. (2016). Effect of phospholipid and ethanol concentrations on physical properties and stability of phenylethyl resorcinol loaded ethosome. *Latin American Journal of Pharmacy*.

[11] Limsuwan T., Amnuaikit T. (2012). Development of ethosomes containing mycophenolic acid. *Procedia Chemistry*.

[12] Jain S., Jain P., Umamaheshwari R. B., Jain N. K. (2003). Transfersomes—a novel vesicular carrier for enhanced transdermal delivery: development, characterization, and performance evaluation. *Drug Development and Industrial Pharmacy*.

[13] Limsuwan T., Songkram C., Amnuaikit T. (2012). In vitro skin permeation study of ethosome containing mycophenolic acid. *Isan Journal of Pharmaceutical Sciences*.

[14] Dej-adisai S., Meechai I., Puripattanavong J., Kummee S. (2014). Antityrosinase and antimicrobial activities from thai medicinal plants. *Archives of Pharmacal Research*.

[15] Tomita Y., Maeda K., Tagami H. (1992). Melanocyte-stimulating properties of arachidonic acid metabolites: possible role in postinflammatory pigmentation. *Pigment Cell Research*.

[16] Barry J. A., Gawrisch K. (1994). Direct NMR evidence for ethanol binding to the lipid-water interface of phospholipid bilayers. *Biochemistry*.

[17] Bendas E. R., Tadros M. I. (2007). Enhanced transdermal delivery of salbutamol sulfate via ethosomes. *AAPS Pharmaceutical Sciences and Technology*.

[18] Maurya S. D. (2010). Enhanced transdermal permeation of indinavir sulfate through stratum corneum via. Novel permeation enhancers: ethosomes. *Der Pharmacia Lettre*.

[19] Dave V., Kumar D., Lewis S., Paliwal S. (2010). Ethosome for enhanced transdermal drug delivery of aceclofenac.. *International Journal of Drug Delivery*.

[20] Rakesh R., Anoop K. R. (2012). Formulation and optimization of nano-sized ethosomes for enhanced transdermal delivery of cromolyn sodium. *Journal of Pharmacy and Bioallied Sciences*.

[21] Honary S., Zahir F. (2013). Effect of zeta potential on the properties of nano-drug delivery systems—a review (Part 2). *Tropical Journal of Pharmaceutical Research*.

[22] Liu J., Hu G. (2007). Advances in studies of phospholipids as carriers in skin topical application. *Journal of Nanjing Medical University*.

[23] Bhalaria M. K., Naik S., Misra A. N. (2009). Ethosomes: a novel delivery system for antifungal drugs in the treatment of topical fungal diseases. *Indian Journal of Experimental Biology*.

[24] Komatsu H., Okada S. (1995). Ethanol-induced aggregation and fusion of small phosphatidylcholine liposome: participation of interdigitated membrane formation in their processes. *BBA - Biomembranes*.

[25] Girhepunje K., Pal R., Gevariya H., Behera A., Thirumoorthy N. (2010). A novel vesicular carrier for enhanced dermal delivery of ciclopirox olamine. *Der Pharmacia Lettre*.

[26] D'Agostino R. B., Sullivan L., Massaro J. Validation of Analytical Procedures: Text and Methodology *Q2 (R1)*.

[27] Higaki K., Amnuaikit C., Kimura T. (2003). Strategies for overcoming the stratum corneum: chemical and physical approaches. *American Journal of Drug Delivery*.

[28] Williams A. C., Barry B. W. (2004). Penetration enhancers. *Advanced Drug Delivery Reviews*.

[29] Dubey V., Mishra D., Jain N. K. (2007). Melatonin loaded ethanolic liposomes: physicochemical characterization and enhanced transdermal delivery. *European Journal of Pharmaceutics and Biopharmaceutics*.

[30] David S. R. N., Hui M. S., Pin C. F., Ci F. Y., Rajabalaya R. (2013). Formulation and in vitro evaluation of ethosomes as vesicular carrier for enhanced topical delivery of isotretinoin. *International Journal of Drug Delivery*.

